# The role of cGAS-STING pathway ubiquitination in innate immunity and multiple diseases

**DOI:** 10.3389/fimmu.2025.1522200

**Published:** 2025-02-14

**Authors:** Chunyan Deng, Dongyan Chen, Liang Yang, Yubiao Zhang, Cheng Jin, Yue Li, Qihong Lin, Mingjing Luo, Ruihao Zheng, Baozhen Huang, Sixi Liu

**Affiliations:** ^1^ Department of Hematology and Oncology, Shenzhen Children ‘s Hospital, Shenzhen, China; ^2^ Shenzhen Institute of Advanced Technology, Chinese Academy of Sciences, Shenzhen, China; ^3^ Department of Orthopedics, Renmin Hospital of Wuhan University, Wuhan, China; ^4^ Department of Biomedical Sciences, City University of Hong Kong, Kowloon, Hong Kong SAR, China

**Keywords:** CGAS, STING, ubiquitination, innate immunity, cancer

## Abstract

The cGAS-STING pathway is essential in innate immunity, especially in antiviral responses and cellular stress management. cGAS acts as a cytoplasmic DNA sensor by initiating the synthesis of the second messenger cyclic GMP-AMP synthase (cGAMP), which subsequently activates the STING pathway, leading to the production of type I interferons and other cytokines, as well as the activation of inflammatory mediators. Recent studies have demonstrated that ubiquitination changes closely regulate the function of the cGAS-STING pathway. Ubiquitination modifications influence the stability and activity of cGAS and STING, while also influencing the accuracy of the immune response by adjusting their degradation and signal intensity. E3 ubiquitin ligase specifically facilitates the degradation or modulates the signaling of cGAS-STING-associated proteins via ubiquitination alterations. Furthermore, the ubiquitination of the cGAS-STING pathway serves distinct functions in various cell types and engages with NF-κB, IRF3/7, autophagy, and endoplasmic reticulum stress. This ubiquitin-mediated regulation is crucial for sustaining the balance of innate immunity, while excessive or inadequate ubiquitination can result in autoimmune disorders, cancers, and viral infections. An extensive examination of the ubiquitination process within the cGAS-STING pathway elucidates its specific regulatory mechanisms in innate immunity and identifies novel targets for the intervention of associated diseases.

## 1 Introduction

Innate immunity serves as the body’s primary defense mechanism against a variety of pathogens, including bacteria, viruses, fungi, and parasites (1). The classical cGAS-STING signaling pathway is an essential element of the innate immune system and significantly contributes to the body’s defense against pathogen invasion (2). The identification of exogenous or endogenous DNA by cGAS activates the enzyme, resulting in the production of significant quantities of cyclic GMP-AMP synthase (cGAMP) (3). This cGAMP then binds to STING, activating it and promoting the translocation of STING from the endoplasmic reticulum to the Golgi apparatus (4, 5). Thereafter, upon activation, STING recruits TBK1 and subsequently activates IRF3, leading to its dimerization and translocation to the nucleus, where it orchestrates the transcription of type I interferon (IFN) genes. Furthermore, the activation of STING facilitates its interaction with the NF-κB signaling pathway, promoting the nuclear translocation of NF-κB and thereby inducing the expression of pro-inflammatory cytokines, such as IL-1β, TNFα, and IL-6, which collectively augment the immune response (6).

Ubiquitination is essential for the regulation of immune pathways, serving as a post-translational modification process in which ubiquitin molecules alter target proteins within cells via specialized enzymes, including ubiquitin-activating enzymes, conjugating enzymes, ligases, and degrading enzymes (7, 8). This mechanism regulates homeostasis in the body by affecting protein activation, localization, and degradation, thus precisely coordinating immune responses. This coordination enables the host to efficiently address threats while averting pathological overactivation that could lead to autoimmune disorders (9). Recent studies have revealed that the activity of the cGAS-STING pathway is tightly regulated by ubiquitination modifications (10, 11). The review aims to provide a comprehensive overview of the ubiquitination processes involved in the cGAS-STING pathway and their roles in various diseases, providing significant therapeutic insights for autoimmune disorders, cancer and viral infections, among other diseases.

## 2 Ubiquitination of cGAS-STING pathway

### 2.1 Ubiquitination in cellular processes of cGAS

Ubiquitination is crucial for the activation, stability, and function of cGAS, necessitating multiple E3 ubiquitin ligases. K63-linked ubiquitination facilitates signal transduction, whereas K48-linked ubiquitination generally indicates degradation (12). TRIM56 induces Lys335 monoubiquitination of cGAS, leading to a significant increase in its dimerization, DNA-binding activity, and cGAMP production, which is important for cytoplasmic DNA sensing and IFNαβ production to induce anti-DNA viral immunity (13). Recent study has revealed the cGAS-based antiphage signaling system (CBASS) in bacteria, with Cap2 and Cap3 comprising around 39% of the CBASS system (14, 15). Cap2 assembles into a hexameric complex with a high affinity for cGAMP, hence obstructing the cGAS signaling pathway and hindering phage proliferation. This transpires via the establishment of a thioester bond with the C-terminal glycine of cGAS, enabling the binding of cGAS to target proteins similarly to ubiquitin attachment (16). K27-linked polyubiquitination of cGAS is predominantly mediated by RNF185, the first E3 ubiquitin ligase of cGAS, to enhance its enzymatic activity (17). K48-linked ubiquitination of cGAS is a recognition signal for p62-dependent selective autophagic degradation. The induction of TRIM14 by IFN-I accelerates cGAS stabilization by recruiting USP14 to cleave the ubiquitin chain of cGAS at lysine (K)414 (18). On the other hand, The E3 ubiquitin ligase TRIM41 positively regulates cGAMP synthesis by interacting with cGAS and facilitating its monoubiquitination (19). cGAS was initially recognized as a cytoplasmic DNA detector (5). A recent study indicated that nucleosoluble cGAS is essential for the recognition of nuclear-replicating DNA viruses (20). Furthermore, TRIM41 interacts with and ubiquitinates ORF2p, affecting its stability, whereas intranuclear cGAS enhances the binding of ORF2p to TRIM41, hence facilitating TRIM41-mediated degradation of ORF2p and restricting LINE-1 (L1) retrotransposition (21). This could offer a route for future intervention in aging and tumorigenesis. USP15 is a constituent of the deubiquitinating enzymes subfamily of cysteine proteases, which facilitate the removal of ubiquitin from substrates in a ubiquitin-specific manner (22). USP15 activates cGAS in the presence of DNA by two parallel pathways: facilitating cGAS deubiquitination and enhancing its phase separation, which contrasts with conventional cGAS deubiquitination mechanisms, such as those mediated by USP14 (18, 23). Furthermore, the deubiquitinating enzyme USP27X associates with cGAS and eliminates K48-linked polyubiquitinated chains from cGAS, leading to the stability of cGAS (24). Andrea Ablasser et al. clarified the mechanism through which the ubiquitin-proteasome system (UPS) degrades nuclear cGAS, identifying SPSB3 as a substrate receptor that targets cGAS and associates with the cullin-RING ubiquitin ligase 5 (CRL5) complex to ubiquitinate nuclear cGAS, facilitating its subsequent degradation (25).

### 2.2 Ubiquitination in cellular processes of STING

STING serves as a pivotal junction protein within the cGAS-STING pathway. The activation of the STING pathway involves the translocation of STING from the ER to the ER-Golgi intermediate compartment (ERGIC) and then to the Golgi apparatus, a crucial step in initiating its downstream signaling pathway (26). The distribution and function of STING are governed by several E3 ubiquitin ligases that modify STING via ubiquitination (27). TRIM56 enhances K63-linked ubiquitination of STING, facilitating STING dimerization and its accumulation in the Golgi apparatus, thereby recruiting TBK1 and stimulating IFN-1β production (28). TRIM32, RNF115, and mitochondrial E3 ubiquitin ligase 1 (MUL1) promote K63-linked polyubiquitination, hence enhancing the efficient transport of STING and the activation of its downstream pathways (29, 30). Researchers at Shandong University discovered that TRIM10 promotes the ubiquitination of lysine residues 289 and 370 of STING at K27 and K29, thereby facilitating the translocation of STING from the ER to the Golgi, enhancing its aggregation in the Golgi as well as the recruitment of the downstream kinase TBK1 (31). The E3 ubiquitin ligase complex, located in the ER and consisting of AMFR-GP78 and INSIG1, promotes K27 polyubiquitination of STING, recruits TBK1, and triggers IFN production (32). The ubiquitination of STING at the K236 site by RNF144A is crucial for STING translocation and the subsequent control of STING-mediated antiviral responses (33). Conversely, USP21 is a significant deubiquitinating enzyme of STING, which negatively modulates the synthesis of IFN-I triggered by DNA viruses by hydrolyzing the K27/63-linked polyubiquitin chains on STING (34). In recent years, many ubiquitin-like proteins have been identified that ubiquitinate STING proteins. UFL1, the only recognized E3 ubiquitin ligase for UFM1, reduces K48-linked ubiquitination of STING at the Lys338, Lys347, and Lys370 residues by competitively binding with STING and TRIM29, thereby inhibiting its degradation, maintaining STING protein stability, and augmenting the antiviral immune response (35).

**Figure 1:**
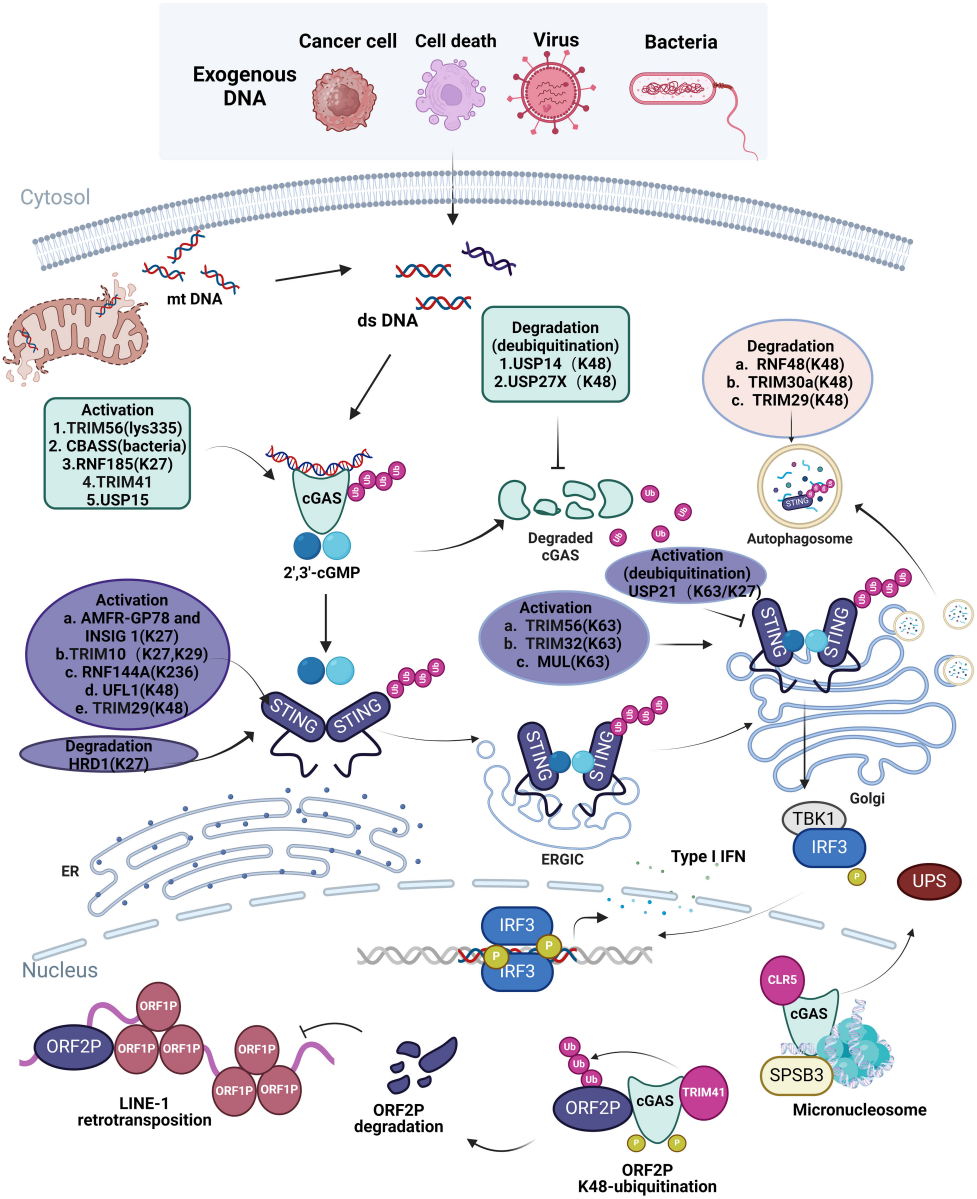
Ubiquitination in cellular processes of cGAS-STING Pathway. Exogenous dsDNA released from cancer cell, cell death, virus, bacteria and endogenous dsDNA released from mitochondria are easily recognized by intracellular cGAS, which promotes the cGAS-STING-TBK1 signaling pathway and releases IFN I to elicit innate immune response. The ubiquitination of different ubiquitinating enzymes to different sites of cGAS and STING in different organelles is listed in the figure; the ubiquitination of cGAS and STING promotes or inhibits the activation and degradation of cGAS and STING, which affects the innate immune response of the cGAS-STING pathway in cells. However, in the nucleus, TRIM41 and CLR5 also cause ubiquitination of cGAS, which restricts L1 retrotransposition as well as degradation of cGAS via the ubiquitin protease hydrolysis system. Image created with BioRender.com, with permission.

RNF5 promotes K48-linked ubiquitination and subsequent degradation of STING, hence suppressing the antiviral immune response (36). Following viral infection, RNF5 is ubiquitinated at the Lys150 site in mitochondria, resulting in the degradation of STING, which inhibits virus-induced signaling (37). RNF5 also restricts IFN-I antiviral responses in herpes simplex virus (HSV) corneal epitheliitis by suppressing STING/IRF3 signaling (37). Conversely, RNF5 and TRIM30a facilitate K48 polyubiquitination of STING, resulting in its proteasomal degradation and suppression of the DNA signaling cascade response (38). TRIM29 serves as a negative-feedback regulator of the intracellular response to DNA and DNA viral stimuli by facilitating K48 ubiquitination of STING, hence increasing its degradation (39). Reverse transport of STING to the ER and translocation of activated STING to the lysosome for degradation are critical pathways for alleviating faulty and excessive STING signaling, where in ubiquitination modifications are significant (40, 41). STING is degraded via a microautophagy mechanism that relies on an endosomal sorting and transport complex (ESCRT). During transport, STING is ubiquitinated at lysine 288 through K63 ubiquitination, a modification that is essential for its degradation to avoid excessive immunization (42). Moreover, HRD1 ubiquitinates STING proteins, mostly through K27-linked ubiquitination, facilitating the degradation of ER-neonatal STING proteins and thereby inhibiting STING-mediated immunological responses (43). These mechanisms highlight the complex regulation of STING by ubiquitination, which is crucial for maintaining the balance of the immune response and preventing excessive or abnormal activation (**Figure 1**).

## 3 Ubiquitination dysregulation of the cGAS-STING pathway is associated with multiple diseases

The dysregulation of ubiquitination in the cGAS-STING pathway is often associated with autoimmune diseases, viral infections, inflammation, and disturbances in intestinal homeostasis. cGAS identifies DNA of a specific length irrespective of its sequence, enabling DNA from any source to provoke an immune response through the activation of the cGAS-STING pathway (44). Hyperactivation of the cGAS-STING pathway contributes to autoimmune illnesses, including rheumatoid arthritis (RA), systemic lupus erythematosus (SLE), and Aicardi-Goutieres syndrome (AGS), as well as other conditions such as cancer (45, 46). Patients with functional mutations in Trex1 trigger autoimmune disorders by persistently stimulating the cGAS-STING signaling pathway due to the accumulation of their own DNA (47). In various DNA viral infections, including Human Immunodeficiency Virus (HIV) and Hepatitis B Virus (HBV), the viral proteins, specifically HIV coat and HBV Pol, disrupt the cGAS-STING signaling pathway by targeting STING ubiquitination, thereby facilitating the modulation of the host immune response and allowing for viral immune evasion to sustain persistent infection in the host (48, 49). The papain-like protease of the RNA virus pig epidemic diarrhea virus and the papain-like protease of coronavirus play significant immunomodulatory effects. They suppress the host immune response by negatively modulating the cGAS-STING pathway, exerting deubiquitination, and exhibiting IFN antagonistic activity, enabling the virus to evade the host’s innate immunological defenses and to persist for replication and transmission (50, 51). Bone marrow chimera investigations indicate that STING accumulation in intestinal macrophages and monocytes serves as a primary instigator of inflammation (11). Modifications in the cGAS-STING DNA signaling pathway influence intestinal homeostasis. Elevated STING expression was identified as a hallmark of intestinal inflammation in mice with colitis and in individuals with inflammatory bowel disease (52). The STING can be triggered by the bacterial product cyclic di-GMP in myeloid cells, leading to K63-linked ubiquitination and subsequent accumulation of STING in intestinal myeloid cells, and then triggering intestinal inflammation (11). Therefore, rigorous regulation of ubiquitination of ubiquitination activation and degradation of cGAS-STING pathway is essential.

## 4 The downstream effects of the ubiquitination process in the cGAS-STING pathway

**Figure 2:**
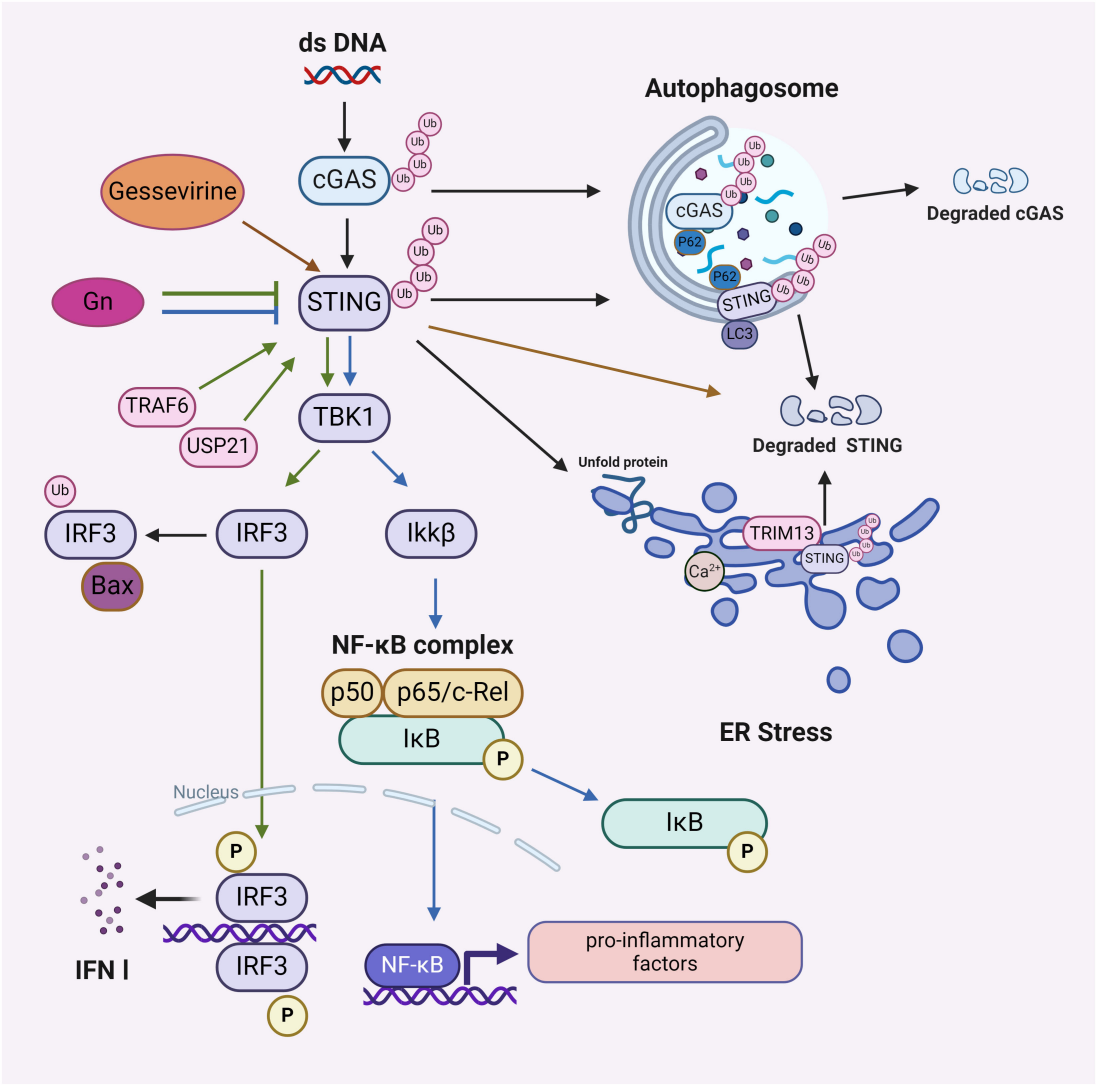
The downstream effects of the ubiquitination process in the cGAS-STING pathway. Affects NF-κB signaling pathway: Gelsevirine affects STING/NF-κB by promoting ubiquitinated degradation of STING and Gn by blocking ubiquitinated activation of STING; affects IRF3/IRF7 signaling pathway: TRAF6 ubiquitinates or USP21 deubiquitinates STING to activate or maintain STING stability, promoting downstream activation of IRF3 and IRF7; affects autophagy: p62 mediates ubiquitination of cGAS and autophagic degradation of STING; affects ER Stress: STING acts as a mediator of ER Stress, and ubiquitination of STING by TRIM13 leads to STING degradation. Image created with BioRender.com, with permission.

The interaction between the ubiquitination of the cGAS-STING pathway and several signaling cascades, such as NF-κB, IRF3/7, autophagy and ER Stress, emphasize the intricacy of immunomodulation (**Figure 2**).

### 4.1 NF-κB

The interplay between the ubiquitination of the cGAS-STING pathway and the NF-κB pathway initiates a cascade that enhances IFN responses and robust host antiviral defenses (53). Gelsevirine, a natural substance, blocks excessive activation of the STING/NF-κB pathway by facilitating TRIM21-mediated K48 ubiquitination degradation of STING to address organ damage resulting from sepsis (54). Furthermore, the envelope glycoprotein Gn of the severe fever with thrombocytopenia syndrome virus (SFTSV) interacts with STING to impede STING dimerization and K27 ubiquitination, thereby hindering the assembly of the STING-TBK1 complex and subsequent signaling, which obstructs the nuclear translocation of IRF3 and p65, ultimately attenuating downstream innate immune signaling (55). This clarifies the connection between the ubiquitination of the cGAS-STING pathway and NF-κB, alongside immunological mechanisms, including enhanced signal activation, viral protein disruption, and modulation by natural products.

### 4.2 IRF3/IRF7

In the context of autoimmune diseases, the ubiquitination of the cGAS-STING pathway can interact with IRF3/IRF7. Aberrant activation of the cGAS-STING pathway may lead to hyperactivation of IRF3/IRF7, thereby eliciting an excessive immune response (56). TRAF6 can ubiquitinate cGAS and the K63 chain of STING to facilitate the subsequent activation of IRF3 and IRF7, hence initiating the production of antiviral INF-I (57, 58). USP21 deubiquitinates STING, enhancing its stability and activity, which then promotes the downstream activation of IRF3 and IRF7, thereby amplifying the antiviral immune response (59). Investigating the interaction mechanism between the ubiquitination of the cGAS-STING pathway and the IRF3/IRF7 pathway may elucidate its significant involvement in autoimmune disorders and antiviral responses.

### 4.3 Autophagy

The cGAS-STING pathway can induce autophagy through the translocation of STING, independently of TBK1-IRF3 and conventional autophagy signaling molecules. Mechanistically, the transfer of STING across membranes and its association with ERGIC-containing membranes are essential for cGAMP-induced autophagy, which effectively eliminates intracellular DNA and viruses (26). STING-dependent signaling network is associated with health and disease by regulating autophagic degradation or various cell death modalities (60). p62 interacts with ubiquitinated STING through its ubiquitin-binding domain, subsequently binding to the autophagosome marker protein LC3, which facilitates the transport of cGAS or STING to the autophagosome for degradation, thereby inhibiting the activation of the cGAS-STING pathway and averting excessive immune responses (61). However, STING activation negatively influences the autophagy process initiated by energy deficiency. The mechanism entails STING binding to STX17, an essential protein for autophagic membrane fusion, which affects the transport of STX17 from the ER to the intact autophagosome, thereby inhibiting the efficiency of autophagosome-lysosome fusion and downregulating cellular autophagy (62). In summary, multiple modes of ubiquitination occur in the cGAS-STING pathway, which binds to the autophagic system to regulate the immune response and maintain cellular homeostasis in organisms.

### 4.4 ER stress

STING was identified as a mediator of ER stress and the UPR through a novel pattern referred to as “the UPR motif” (63). Additionally, the cGAS-STING pathway may induce ER stress through an interaction between STING and the calcium sensor stromal interaction molecule 1 (STIM1) (64). Given that TRIM13 is consistently localized in the ER, it may modulate STING degradation via ER-mediated degradation (65, 66). This may differ from the ERGIC-initiated autophagosome route and the post-ER-initiated autophagy pathway (26). A proposed strategy for regulating STING homeostasis during inflammatory responses induced by pathogenic DNA involves the transmembrane ER-associated TRIM13. This ER-localized E3 ubiquitin ligase TRIM13 interacts with STING through transmembrane structural domains and facilitates the polyubiquitylation of STING’s Lys6 ligand, leading to a reduction in ER exit and an increase in ER-mediated STING degradation (67).

## 5 Therapeutic potential on targeting ubiquitination in the cGAS-STING pathway

Targeting the ubiquitination of the cGAS-STING pathway holds significant potential in the treatment of autoimmune diseases, cancer, and viral infections. In autoimmune diseases like SLE, the overactivation of the cGAS-STING pathway can lead to excessive immune responses, with RNF185 being a key E3 ubiquitin ligase implicated in SLE pathogenesis (17). The SARS-CoV-2 PLPRO removes polyubiquitin chains from STING, inhibiting IFN-I responses and providing insights into viral interactions with the cGAS-STING pathway (68). Additionally, the deubiquitinating enzyme MYSM1 interacts with and modifies STING, preventing downstream signaling and offering new therapeutic avenues (69). In cancer research, the cGAS-STING pathway is significant for tumor development and therapy. Activating this pathway can enhance the ‘tumor-immune’ arousal effect, improving chemotherapy sensitivity. IDI1, a metabolic enzyme, interacts with cGAS, and TRIM41, an E3 ligase from hepatocellular carcinoma cells, promotes cGAS degradation, suggesting a role in innate immunity and a potential target for liver cancer treatment (70). TRIM29 induces STING degradation, affecting DNA viral infections, and its knockdown in airway epithelial cells enhances INF-I production, nearly eradicating EBV in nasopharyngeal carcinoma cells (39). Meanwhile, the deubiquitinating enzyme USP35 is a negative regulator of STING-associated INF-I signaling in ovarian cancer, with its silencing triggering potent anti-tumor activity and improving prognosis (71). Furthermore, TRIM29-mediated STING ubiquitination degrades STING in immune and cancer cells, and its overexpression hinders immune responses. The protein-protein interaction modulator SB24011 inhibits STING-TRIM29, upregulating cellular STING levels and showing potential as an anti-cancer therapy (72). Targeting the DTX3L-cGAS axis may also be a promising approach for pancreatic tumor treatment (73). Developed by Professor Jin Jian and Professor Wenyi Wei, the first-in-class deubiquitinase-targeting chimeras (DUBTACs) of cGAS MS7829 and MS8588 stabilize and activate cGAS, effectively inhibiting cancer cell growth by enhancing the cGAS-STING signaling pathway (74). The cGAS-STING pathway is also a key in viral infections, with TRIM30α induced by HSV-1 infection in dendritic cells, promoting STING degradation and acting as a negative feedback regulator of the innate immune response (75, 76). MARCH8 negatively regulates the cGAS-mediated natural immune signaling pathway, and ARIH1 promotes antiviral and autoimmunity responses by catalyzing mono-ISGylation and cGAS oligomerization (77, 78). Knockdown of TRIM14 impairs the antiviral response triggered by HSV-1 in a cGAS-dependent manner (18). These findings highlight the complexity of the cGAS-STING pathway in targeting ubiquitination in viral infections, offering potential research directions and therapeutic targets (79). Targeting the ubiquitination of the cGAS-STING pathway holds significant potential in the treatment of autoimmune diseases, cancer, and viral infections.

## 6 Discussion

Ubiquitination alterations of the cGAS-STING pathway are essential for its functionality and stability. The E3 ubiquitin ligases TRIM41 and TRIM56 augment dimerization, DNA-binding activity, and cGAMP synthesis; RNF185, and the deubiquitinating enzymes USP15 and USP27X promote cGAS stability (13, 17, 19, 23, 24). TRIM32, TRIM56, MUL1, and TRIM10 ubiquitin ligases facilitate the translocation of STING from the endoplasmic reticulum to the Golgi, augment its retention in the Golgi, and enlist TBK1 along with subsequent pathways for INF-I synthesis (28–31). Further, many ubiquitinating enzymes are present to facilitate the degradation of STING (37, 75). The interaction between the ubiquitination of the cGAS-STING pathway and other signaling cascades underscores the intricacy of immune control. Ubiquitinated cGAS or STING can be identified by the autophagy receptor protein p62/SQSTM1, which subsequently directs autophagosomes for destruction (40). The natural compound Gelsevirine prevents excessive activation of the STING/NF-κB pathway by facilitating the ubiquitin-mediated degradation of STING (54). The ubiquitination modification of the cGAS-STING pathway interacts with the IRF3/7 pathway, influencing antiviral and autoimmune responses. Furthermore, the cGAS-STING pathway may elicit ER stress via the interaction of STING with the calcium sensor STIM1 (64). SLE, the E3 ubiquitin ligase RNF185 induces hyperactivation of the pathway associated with autoimmune disorders (17). Viral proteins in infections influence host immunological responses and facilitate immune evasion by disrupting STING ubiquitination (68). In addition, in pancreatic tumors, ovarian cancer, and nasopharyngeal carcinoma, blocking the ubiquitination of the cGAS-STING pathway may represent a novel therapeutic approach to inhibit anti-tumor immunity (39, 70, 74).

In summary, the cGAS-STING pathway is crucial for innate immunity, with ubiquitination changes meticulously regulating its function, hence influencing the stability, activity, and accuracy of the immunological responses of cGAS and STING. The cGAS-STING pathway functions differently across many cell types and engages with multiple pathways; its dysregulation can result in numerous illnesses. Focusing on the particular ubiquitin ligases and deubiquitinating enzymes implicated in the cGAS-STING pathway presents a promising approach for the creation of therapeutics for autoimmune diseases, cancer, and viral infections, highlighting the necessity of investigating these interactions for novel therapeutic targets.
